# Profiles of Family and Friend Caregivers of Community-Dwelling People With Dementia: A Latent Class Analysis

**DOI:** 10.1093/geroni/igaa057.191

**Published:** 2020-12-16

**Authors:** Eric Jutkowitz, Lauren Mitchell, Joseph Gaugler

**Affiliations:** 1 Brown University, Providence, Rhode Island, United States; 2 Minneapolis VA, Minneapolis, Minnesota, United States; 3 University of Minnesota, Minneapolis, Minnesota, United States

## Abstract

People living with Alzheimer’s disease and related dementias (ADRD) receive most of their care from family/friends, but little is known about the organization of this care. We used data from the Health and Retirement Study and latent class analysis to determine variation in the hours of care received by community-dwelling people with ADRD from disease onset up to 6-years post onset. At incidence (n=1,158), the latent class analysis identified two groups of caregiving patterns. In the first group, 10% (n=109) of people with ADRD received 481 hours (SD=177) of care. Most care was provided by a spouse (411 hours) with less from children (28 hours), other family/friends (17 hours), and paid individuals (25 hours). In the second latent class, the remaining 90% (n=1,049) of people with ADRD received 114 hours (SD=202) of care which was distributed between spouses (12 hours), children (51 hours), other relatives/friends (22 hours), and paid individuals (29 hours). By 6-years post incidence, 7% (n=76) of the original ADRD cohort remained in the community, and we identified two latent classes independent of those identified at incidence. Almost 15% (n=11) of people with ADRD received a majority of care from a spouse (376 hours) with care supplemented by children (10 hours) and paid individuals (54 hours). The remaining 85% (n=65) of people with ADRD received 294 (SD=314) hours of care from spouses (13 hours), children (104 hours), other family/friends (83 hours), and paid individuals (67 hours). Policies/interventions supporting caregivers must account for the heterogeneity in the organization caregivers.

